# Association of Vegetable and Animal Flesh Intake with Inflammation in Pregnant Women from India

**DOI:** 10.3390/nu12123767

**Published:** 2020-12-08

**Authors:** Su Yadana, Sameera A. Talegawkar, Jyoti S. Mathad, Mallika Alexander, Kripa Rajagopalan, Pavan Kumar, Shilpa Naik, Cheng-Shiun Leu, Vandana Kulkarni, Prasad Deshpande, Mariana Araujo-Pereira, Ramesh Bhosale, Subash Babu, Bruno B. Andrade, Laura E. Caulfield, Amita Gupta, Rupak Shivakoti

**Affiliations:** 1 Department of Epidemiology, Columbia University Mailman School of Public Health, New York, NY 10032, USA; sy2821@cumc.columbia.edu; 2 EcoHealth Alliance, New York, NY 10018, USA; 3 Departments of Exercise and Nutrition Sciences and Epidemiology, Milken Institute School of Public Health, The George Washington University, Washington, DC 20052, USA; talega1@email.gwu.edu; 4 Department of Medicine, Weill Cornell Medical College, New York, NY 10065, USA; jsm9009@med.cornell.edu; 5 Byramjee-Jeejeebhoy Government Medical College-Johns Hopkins University Clinical Research Site, Pune 380016, India; mallika.alexander@yahoo.com (M.A.); vandanakulkarni_5@hotmail.com (V.K.); prass87@gmail.com (P.D.); agupta25@jhmi.edu (A.G.); 6 Division of Nutritional Sciences, Cornell University, Ithaca, NY 14850, USA; kr489@cornell.edu; 7 Department of Medicine, Johns Hopkins School of Medicine, Baltimore, MD 21205, USA; 8 International Center for Excellence in Research, National Institutes of Health, National Institute for Research in Tuberculosis, Chennai 600031, India; pavanmedtech@gmail.com (P.K.); subbaraman.babu@nih.gov (S.B.); 9 Department of Obstetrics and Gynecology, Byramjee Jeejeebhoy Government Medical College, Pune 380016, India; shilunnaik@yahoo.co.in (S.N.); drrameshbhosale@gmail.com (R.B.); 10 Department of Biostatistics, Columbia University Mailman School of Public Health, New York, NY 10032, USA; cl94@cumc.columbia.edu; 11 Instituto Goncalo Moniz, Fundação Oswaldo Cruz, Salvador 40296-710, Brazil; araujopereira.mariana@gmail.com (M.A.-P.); bruno.andrade@fiocruz.br (B.B.A.); 12 Multinational Organization Network Sponsoring Translational and Epidemiological Research, Salvador 45204-040, Brazil; 13 Faculdade de Medicina, Universidade Federal da Bahia, Salvador 40110-100, Brazil; 14 Curso de Medicina, Faculdade de Tecnologia e Ciências, Salvador 41741-590, Brazil; 15 Universidade Salvador (UNIFACS), Laureate Universities, Salvador 41720-200, Brazil; 16 Escola Bahiana de Medicina e Saúde Pública (EBMSP), Salvador 40290-000, Brazil; 17 Center for Human Nutrition, The Johns Hopkins Bloomberg School of Public Health, Baltimore, MD 21205, USA; lcaulfi1@jhu.edu

**Keywords:** vegetable intake, meat intake, monocyte activation, gut barrier, inflammation, pregnancy

## Abstract

In pregnant women, studies are lacking on the relationship of vegetable and animal flesh (poultry, red meat and seafood) intake with inflammation, especially in low- and middle-income countries. We conducted a cohort study of pregnant women receiving antenatal care at BJ Medical College in Pune, India. The dietary intake of pregnant women was queried in the third trimester using a validated food frequency questionnaire. Twelve inflammatory markers were measured in plasma samples using immunoassays. Only 12% of the study population were vegetarians, although animal flesh intake levels were lower compared to Western populations. In multivariable models, higher intakes of total vegetables were associated with lower levels of the T-helper (Th) 17 cytokine interleukin (IL)-17a (*p* = 0.03) and the monocyte/macrophage activation marker soluble CD163 (sCD163) (*p* = 0.02). Additionally, higher intakes of poultry were negatively associated with intestinal fatty-acid binding protein (I-FABP) levels (*p* = 0.01), a marker of intestinal barrier dysfunction and Th2 cytokine IL-13 (*p* = 0.03), and higher seafood was associated with lower IL-13 (*p* = 0.005). Our data from pregnant women in India suggest that a higher quality diet emphasizing vegetables and with some animal flesh is associated with lower inflammation. Future studies should confirm these findings and test if modulating vegetables and animal flesh intake could impact specific aspects of immunity and perinatal health.

## 1. Introduction

The relationship between high inflammation and adverse health outcomes is well-documented [1,2]. Data from the past few decades show that unresolved inflammation is central to many unfavorable disease outcomes, including cardiovascular disease, diabetes and arthritis [1,2]. Inflammation during pregnancy is also a known risk factor for adverse perinatal outcomes such as miscarriage and preterm birth [3,4,5,6]. The balance between pro- and anti-inflammatory immune responses during pregnancy is a critical factor in birth and neonatal outcomes, where the immune system needs to respond to pathogens without harming the fetus [7]. Although there are differences in inflammation by gestational age, an excess pro-inflammatory immune response in mid- to late-pregnancy has been associated with preterm birth (PTB) [3,4,5,6]. Studying maternal factors during pregnancy, such as diet, that can impact inflammation is an area of active research and might help identify modifiable factors that can improve perinatal health.

The relationship of dietary components and specific inflammatory markers has been extensively studied in non-pregnant adults from Western countries. In studies focused on vegetable and animal flesh food intake, data generally show that diets with higher intakes of fruits, vegetables and fibers, along with lower intakes of animal flesh (especially red meat), are associated with reduced inflammation in circulation [8,9,10,11,12,13]. While similar relationships have also been observed in pregnant populations [14,15], these studies have mainly focused on acute phase proteins (e.g., C-reactive protein (CRP)) and general inflammatory cytokines (e.g., IL-6, IL-1β, tumor necrosis factor α (TNFα)), with a lack of data regarding markers that inform on other aspects of immunity.

For instance, we and others have shown that higher circulating concentrations of immune markers for gut epithelial barrier dysfunction (e.g., intestinal fatty acid binding protein (I-FABP)) and monocyte activation (soluble CD14 (sCD14) and CD163 (sCD163)) are associated with PTB in specific populations [16,17]. Similarly, the balance of T-helper (Th) cells and cytokines related to Th1, Th2 and Th17 immune responses can impact pregnancy outcomes [14,18]. Whether and how vegetable, fruit and animal flesh intake is associated with these markers of gut integrity, monocyte activation or Th cytokines has not been studied, especially in the context of pregnancy. Furthermore, studies on diet and inflammation are lacking in resource-limited settings [10], where the diet and immune profile (e.g., more gut barrier dysfunction) is unique [19].

To address these research gaps, the objective of this study was to determine the association of vegetable and animal flesh intake with systemic markers that inform on various aspects of immunity, including gut integrity, monocyte activation, Th-associated cytokines (Interferon (IFN)-γ) for Th1, IL-13 for Th2 and IL-17a for Th17), along with acute phase response, and other general inflammatory markers (e.g., IL-6, IL-1β  and IFN-β) in pregnant women from India.

## 2. Materials and Methods 

### 2.1. Study Design and Population 

We conducted a cohort study of pregnant women receiving antenatal care at Sassoon Hospital/Byramjee Jeejeebhoy Government Medical College (BJGMC) in Pune, India [20]. The primary objective of the cohort study was to determine the temporal differences in immunity among pregnant women with and without latent Tuberculosis infection (LTBI). The cohort study was stratified by human immunodeficiency virus (HIV) and LTBI status, and used convenience sampling to screen and enroll pregnant women at BJGMC. Women who were between 13 and 34 weeks of gestation, who met eligibility criteria and provided informed consent, were selected for this cohort study [20]. In this analysis, we evaluated the associations of vegetable, fruit and animal flesh intake during pregnancy with maternal systemic inflammation among all women with available data and samples from the parent cohort study.

### 2.2. Ethics Statement

The study was conducted in accordance with the Declaration of Helsinki, and the protocol was approved by the ethics committee of BJGMC (IRB 00003631), Johns Hopkins University (IRB 00068973), Weill Cornell Medicine (IRB 1503016041) and Columbia University (IRB AAAR9863). We followed guidelines for human experimentation from the US Department of Health and Human Services.

### 2.3. Data Collection 

Participants were enrolled at either the second or third trimester, and follow-up visits were conducted at the third trimester (for those who were enrolled at the second trimester), delivery, 6 weeks postpartum and every 3 months after delivery up to a year post-partum. Relevant data including clinical history (LTBI, gestational diabetes and HIV status) and anthropometric status (mid-upper arm circumference, MUAC) were collected at enrollment and follow-up visits. Undernutrition was defined based on a MUAC cutoff of <23 cm and obesity with a MUAC cutoff of >30.5 cm [21]. We also collected sociodemographic information including age, education and smoking status using a questionnaire administered to pregnant women during enrollment and subsequent visits and operationalized as detailed in Table 1.

We queried the dietary intake of pregnant women using Nutrition Assistant-Diet in India Study of Health (NINA-DISH), a tablet-based, interviewer-administered semi-quantitative food-frequency questionnaire (FFQ) developed by Daniel et al. to measure diet and nutrient profiles across diverse populations of India [22]. We adapted this FFQ and then evaluated its validity for our pregnant study population in Pune [23]. It was administered during the third trimester and queried usual dietary intake from the second trimester through to the interview. As per the original NINA-DISH FFQ, dietary intake data were linked to food composition databases from 7 sources (United Kingdom, Food and Nutrient Database for Dietary Studies (FNDDS), Singapore, Malaysia, Nutrient content of food in Australia (NUTTAB), United States Department of Agriculture (USDA), and WorldFood) [22,23,24,25,26,27,28,29]. The output variables included intakes of energy, macronutrients and micronutrients, as well as intakes of foods and food groups.

### 2.4. Laboratory Assessments

Blood samples were collected from pregnant women at the third trimester visit in heparin tubes and plasma was extracted and stored at −80 °C until further use. Inflammation markers assessed in this study were markers of intestinal barrier dysfunction I-FABP, monocyte activation sCD14 and sCD163, Th1 cytokines (interferon gamma (IFN-γ) and tumor necrosis factor (TNF-α)), Th2 cytokine (IL-13), Th17 cytokine (IL-17a), acute phase proteins (CRP and α-1-glycoprotein (AGP)) and other pro-inflammatory cytokines (IL-6, inflammasome activation marker interleukin 1β (Il-1β) and type I interferons (IFN-β)). As described above, we selected these markers based on their importance to pregnancy immunology and perinatal outcomes (e.g., PTB) [16,17]. It is worth noting that while these cytokines are important players in the specific immune functions we have attributed to them above, many of the cytokines also have additional functions; for example, while IFNγ and TNFα are important in Th1 responses, they are also produced by other cell types and levels in circulation might reflect immune responses not related to Th1 responses. Luminex multiplex enzyme-linked immunosorbent assay (ELISAs) purchased from R&D Systems (Minneapolis, USA) was used to measure plasma concentrations of IFN-γ, IL-13, IL-17a, TNF-α, IL-1β and IL-6. Single-plex ELISAs purchased from R&D Systems (Minneapolis, USA) were used to measure plasma CRP, AGP, I-FABP, IFN-β, sCD163 and sCD14. The samples were run in duplicates at the National Institute of Health—National Institute for Research in Tuberculosis—International Center for Excellence in Research laboratory at Chennai, India.

### 2.5. Statistical Analyses

Univariable and multivariable linear regression analyses were used to determine the association of vegetables, fruits and animal flesh intakes with maternal inflammation markers. For this analysis, the outcome variables were concentrations of the various inflammatory markers. The exposure variables were grams/day of (i) vegetable + fruit intake, and (ii) animal flesh food (poultry + red meat + seafood) intake. We further assessed sub-categories of exposure variables: (1a) vegetables, (1b) fruits, (2a) poultry, (2b) red meat and (2c) seafood. We also assessed further sub-categories of vegetables, including dry vegetables, green vegetables and raw/salad vegetables, and the specific vegetables within each sub-category are detailed in Supplementary Table S1.

Maternal plasma concentrations were log_2_-transformed, and food group intake variables were log_e_-transformed. Pregnant women with implausible energy intakes, defined based on prior studies [23,30] as daily total energy take of <500 or >4800 kcal, were excluded from analyses. For the animal flesh analysis, in order to analyze log-transformed continuous data, we further excluded individuals with zero values for intakes of animal flesh food groups (i.e., poultry, red meat or seafood). This included lacto-ovo-vegetarians (n = 23) and those that were not lacto-ovo-vegetarians but who only consumed animal flesh as parts of food categorized in other food groups (n = 6) (e.g., chicken biryani categorized under cereals). Of note, we also conducted additional analysis for animal flesh intake with a categorical exposure variable that included a category for those who did not consume animal flesh. This analysis was less informative (data not shown), likely due to methodological drawbacks in categorizing continuous variables, including loss of power in studies with a limited sample size [31].

In both univariable and multivariable models, we adjusted for total energy [32]. Potential confounding factors, including age, education, gestational age, smoking status and HIV status, were selected using directed acyclic graphs and were adjusted for in multivariable models. In additional models, we also tested whether our results differed with further adjustments for mother’s MUAC, gestational diabetes, preeclampsia, total fat intake, total vegetables and fruits intake (for animal flesh analyses) and total animal flesh intake (for vegetables and fruit analyses).

All analyses were conducted using STATA/IC version 16.0 (StataCorp., College Station, TX, USA) and a *p*-value < 0.05 was considered statistically significant.

## 3. Results

### 3.1. Study Population and Characteristics 

A total of 246 women were enrolled in the cohort study. After excluding pregnant women without data on inflammation markers (n = 26), and those with missing dietary data (n = 1) or implausible energy intakes (n = 33), 186 women were available for the analysis on the relationship of vegetable and fruit intake with maternal inflammation. Study population characteristics are detailed in Table 1**.** Of note, based on the design of the parent cohort study (i.e., stratified by LTBI and HIV status), about 31.2% of women in this study were HIV+ and 70.4% were LTBI+ (Table 1). Surprisingly, only 12% of the study population were lacto-ovo-vegetarians.

### 3.2. Vegetable/Fruit Intake and Maternal Inflammation

The median (25th percentile, 75th percentile) total vegetables and fruit intake, total vegetables intake, fruits intake, dry vegetables intake, green vegetables intake and salad intake are shown in Table 2. Total vegetable and fruit intake levels were 432.0 (322.5, 602.5) g/day, with vegetable intake at 324.0 (244.4, 450.5) g/day and fruit intake at 79.8 (27.4, 180.4) g/day (Table 2). We also assessed the intake levels among those with the highest (Quartile 4: Q4) and lowest (Q1) levels of total vegetables and fruits consumption. The median intake was 242.2 and 761.3 g/day among individuals at Q1 and Q4, respectively.

**Figure 1:**
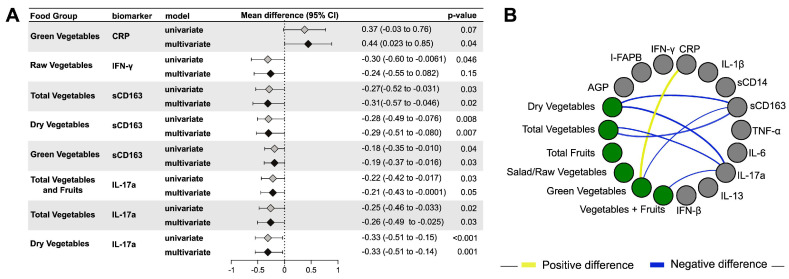
Association of vegetable and fruit intake with markers of inflammation. Using linear regression, the mean difference in log_2_ concentration of each inflammation markers and 95% confidence intervals (95% CI) per unit (log_e_ grams/day) change in total vegetable and fruit intake is shown in Figure 1. We further assessed the relationship with inflammation markers by unit change (log_e_ grams/day) of (a) total vegetables and (b) fruits, along with sub-categories of vegetables: (i) dry vegetables, (ii) green vegetables and (iii) raw/salad vegetables. Only significant results (*p* < 0.05) in univariable or multivariable analyses are shown in the forest plot (Figure 1**A**), with complete data shown in Supplementary Tables S2–S4 and S6–S8. The network graph (Figure 1**B**) shows statistically significant mean differences of inflammatory markers (gray nodes) for each food group (green nodes). Positive or negative mean differences are represented by yellow or blue edges, respectively. The size of the edges is proportional to log_2_ mean differences. Only significant (*p* < 0.05) multivariable models are shown in this chart. Univariable models are adjusted for average daily energy intake and multivariable models are adjusted for daily energy intake, HIV status, age, education, gestational age at sampling and current smoking status.

In univariable linear regression models adjusting for total energy intake only, higher intakes of total vegetables and fruits (mean log_2_ difference: −0.22, 95% confidence intervals (CI): −0.42 to −0.02; *p* = 0.03), total vegetables (mean log_2_ difference: −0.25, 95% CI: −0.46 to −0.03; *p* = 0.02) and dry vegetables (mean log_2_ difference: −0.33, 95% CI: −0.51 to −0.15; *p* < 0.001) were negatively associated with concentrations of the Th17 cytokine IL-17a (Figure 1). Higher intakes of total vegetables (mean log_2_ difference: −0.27, 95% CI: −0.52 to −0.03; *p* = 0.03), dry vegetables (mean log_2_ difference: −0.28, 95% CI: −0.49 to −0.08; *p* = 0.008) and green vegetables (mean log_2_ difference: −0.18, 95% CI: −0.35 to −0.01; *p* = 0.04) were associated with lower concentrations of sCD163, a monocyte activation marker, in univariable models (Figure 1). Surprisingly, we also observed that green vegetables were positively associated with concentrations of CRP (mean log_2_ difference: 0.37, 95% CI: −0.03 to 0.76; *p* = 0.07). In addition, there was a negative association of raw/salad vegetables (mean log_2_ difference: −0.30, 95% CI: −0.60 to −0.006; *p* = 0.046) with IFN-γ in univariable models (Figure 1).

Similar results were observed in multivariable models adjusting for maternal age, education, HIV status, gestational age at interview and smoking status. Higher intakes of total vegetables and fruits (adjusted mean log_2_ difference: −0.21, 95% CI: −0.43 to −0.0001; *p* = 0.05), total vegetables (adjusted mean log_2_ difference: −0.26, 95% CI: −0.49 to −0.025; *p* = 0.03) and dry vegetables (adjusted mean log_2_ difference: −0.33, 95% CI: −0.51 to −0.14; *p* = 0.001) were associated with lower concentrations of IL-17a (Figure 1). Higher intakes of total vegetables (adjusted mean log_2_ difference: −0.31, 95% CI: −0.57 to −0.05; *p* = 0.02), green vegetables (adjusted mean log_2_ difference: −0.19, 95% CI: −0.37 to −0.02; *p* = 0.03) and dry vegetables (adjusted mean log_2_ difference: −0.29, 95% CI: −0.51 to −0.08; *p* = 0.007) were negatively associated with sCD163 concentration. Higher intakes of green vegetables (adjusted mean log_2_ difference: 0.44, 95% CI: 0.02 to 0.85, *p* = 0.04) remained significantly and positively associated with CRP concentration (Figure 1). Surprisingly, we did not observe any significant association of fruit intake with any of these inflammatory markers in univariable or multivariable models.

### 3.3. Animal Flesh Intake and Maternal Inflammation 

For the analysis of animal flesh intake and maternal inflammation, in addition to the exclusion criteria (i.e., missing data or implausible energy intakes) for the vegetable analysis, we also excluded individuals who were lacto-ovo-vegetarians (n = 23) and who did not consume animal flesh even though they were not vegetarian (n = 6), for an analytical sample size of n = 157. The median (25th percentile, 75th percentile) animal flesh (red meat + poultry + seafood) intake, red meat intake, poultry intake and seafood intake are 74.4 (45.4, 135.8) g/day, 37.7 (17.6, 50.3) g/day, 47.0 (23.5, 50.4) g/day and 31.5 (13.7, 54.2) g/day, respectively (Table 2). The median animal flesh intake was 23.5 and 168.0 g/day among individuals at Q1 and Q4, respectively.

**Figure 2:**
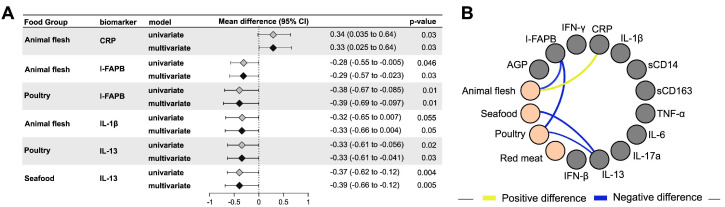
Association of animal flesh intake with markers of inflammation.Using linear regression, Table 2. concentration of each inflammation markers and 95% CI per unit (log_e_ grams/day) change in total animal flesh (poultry + red meat + seafood) intake is shown in Figure 2. We further assessed the relationship of inflammation markers by unit change (log_e_ grams/day) of sub-categories of animal flesh: (i) poultry, (ii) red meat and (iii) seafood. Only significant results (*p* < 0.05) in univariable or multivariable analyses are shown in the forest plot (Figure 2**A**), with complete data shown in Supplementary Tables S5 and S9–S11. The network graph (Figure 2**B**) shows statistically significant mean differences of inflammatory markers (gray nodes) for each food group (green nodes). Positive or negative mean differences are represented by yellow or blue edges, respectively. The size of the edges is proportional to log_2_ mean differences. Only significant (*p* < 0.05) multivariable models are shown in this chart. Univariable models are adjusted for average daily energy intake and multivariable models are adjusted for daily energy intake, HIV status, age, education, gestational age at sampling and current smoking status.

In univariable regression models adjusting for total energy intake only, intakes of animal flesh (mean log_2_ difference: −0.28 95% CI: −0.55 to −0.005; *p* = 0.046), and poultry (mean log_2_ difference: −0.38, 95% CI: −0.67 to −0.09; *p* = 0.01) were negatively associated with I-FABP, a marker for intestinal barrier dysfunction (Figure 2). Higher intakes of poultry (mean log_2_ difference: −0.33, 95% CI: −0.61 to −0.06; *p* = 0.02) and seafood (mean log_2_ difference: −0.37, 95% CI: −0.62 to −0.12; *p* = 0.004) were associated with lower concentrations of the Th2 cytokine IL-13 in univariable models (Figure 2). Higher intakes of animal flesh (mean log_2_ difference: −0.32, 95% CI: −0.65 to 0.007; *p* = 0.05) were associated with lower concentrations of inflammasome activation marker IL-1β. Higher intakes of animal flesh (mean log_2_ difference: 0.34, 95% CI: 0.04 to 0.64; *p* = 0.03) were associated with higher concentrations of CRP.

Similar results were observed in multivariable models adjusting for maternal age, education, HIV status, gestational age at interview and smoking status. Higher intakes of animal flesh (adjusted mean log_2_ difference: −0.29, 95% CI: −0.57 to −0.02; *p* = 0.03), and poultry (adjusted mean log_2_ difference: −0.39, 95% CI: −0.69 to −0.10; *p* = 0.01) remained negatively associated with I-FABP concentrations (Figure 2). Higher intakes of poultry (adjusted mean log_2_ difference: −0.33, 95% CI: −0.61 to −0.04; *p* = 0.03) and seafood (adjusted mean log_2_ difference: −0.39, 95% CI: −0.66 to −0.12; *p* = 0.005) also remained negatively associated with IL-13 concentration (Figure 2). Higher intakes of animal flesh (adjusted mean log_2_ difference: −0.33, 95% CI: −0.66 to 0.004; *p* = 0.055) were negatively associated with IL-1β concentrations and positively associated with CRP concentrations (adjusted mean log_2_ difference: 0.33, 95% CI: 0.03 to 0.64; *p* = 0.03) (Figure 2). Our results for both vegetable intake and animal flesh intake did not change (data not shown) when we further adjusted for preeclampsia, gestational diabetes, LTBI, MUAC, fat intake per day, animal flesh intake per day (for vegetables intake exposure) and vegetables and fruits intake per day (for animal flesh intake exposure).

## 4. Discussions

In our study of pregnant women, we assessed the relationship of vegetable and animal flesh intake with inflammation during the third trimester. Surprisingly, only 12% of our Indian study population were vegetarians, although those who consumed animal flesh had lower levels of intakes compared to the levels reported in Western countries [33,34]. Furthermore, distinct from Western countries, most of the animal flesh consumed was poultry, and red meat was mostly mutton. In the context of this dietary profile, our overall results suggest that a diet higher in vegetables and with some poultry and seafood intake, in a population with low overall animal flesh intake, is associated with lower levels of inflammatory markers involved in gut barrier dysfunction, monocyte activation, and Th2 and Th17 cytokines. Future larger studies should confirm these relationships, many of which are novel observations in pregnant populations. Given that other diets, such as the Mediterranean diet or the Dietary Approaches to Stop Hypertension (DASH) diet, that also highlight similar properties (e.g., more vegetables, lower red meat and increased seafood) have suggested a lower risk of preterm delivery [35,36], further studies should also test whether modifying overall dietary pattern or alternatively a more directed dietary intervention (e.g., increased vegetable intake) could reduce specific markers of inflammation (e.g., sCD163 and IL-17a) and improve pregnancy outcomes.

In our study, we were surprised to find that only 12% of the study population were vegetarians. We think our study population characteristics including low-income urban women, religion (~30% non-Hindu) along with the rapid nutrition transition (where previously vegetarian Hindu and Jain are also consuming animal flesh more commonly) happening overall in India help explain this.

Related to vegetables, we showed that higher intakes of vegetables, and more specifically dry vegetables, were associated with lower concentrations of IL-17, a Th17 cytokine. Studies of Th17 cells in pregnancy show that increased concentrations of Th17 cells and the IL-17a cytokine are linked to adverse pregnancy outcomes, such as miscarriage and preterm birth [18,37,38,39]. There are limited studies, and to our knowledge there are no studies in pregnant populations, that have assessed the relationship of vegetable intake and circulating concentrations of IL-17a. In a study of children, results indicated that frequent consumption of vegetables and grains were associated with lower concentrations of IL-17f [40], another cytokine which, along with IL-17a, is produced by activated Th17 cells. The pathways through which vegetable intake influences IL-17a concentration require further study, but one potential reason could be due to quercetin, a flavanol that is present in fruits and vegetables [41], which has been shown to inhibit IL-17 production [42]. Future studies should confirm whether increased vegetable intake, particularly dry vegetables, might be beneficial in inhibiting the activation of Th17 responses to help maintain a successful pregnancy.

Our result show that higher intakes of vegetables, both green and dry vegetables, were inversely associated with sCD163, a marker of monocyte/macrophage activation. sCD163 plays an important role in pregnancy, as higher concentrations of sCD163 and monocyte activation in general have been linked to adverse pregnancy outcomes, including preeclampsia, gestational diabetes and preterm birth [16,17,43,44]. There are very limited data on the impact of vegetable intake on sCD163 specifically or monocyte activation in general. A study in non-pregnant adults in Taiwan reported that a dietary pattern, which was partly characterized by high intake of vegetables was protective against elevated sCD163 [45]. The reason for this potential relationship is not clear but one proposed pathway through which vegetable intakes might be protective against monocyte/macrophage-mediated inflammation is through red blood cell (RBC) function [45]. High-fat diets have been shown to modulate the activation of CD163+ macrophages through increased RBC aggregation and alteration in RBC membrane components [46,47]. In contrast consumption of green and leafy vegetables has been shown to improve RBC function [48], which may ultimately reduce monocyte/macrophage activation. To our surprise, higher green vegetables intakes was associated with higher concentrations of CRP. The reasons for this are not clear, and we did not observe differences in green vegetables intake by HIV status, LTBI status or smoking status of these women. This requires further study, including whether environmental toxins (e.g., pesticides) in vegetables, and especially dry vegetables, may explain this relationship. In our study, we did not find any significant association of fruit intake with inflammatory markers. This could be partly explained by the low fruit intake (74.4 g/day) in our study population compared to the WHO guidelines of 200 g/day [49].

Our result of positive association between overall animal flesh and acute phase protein CRP is consistent with other studies [50,51]. Most of the studies assessing animal flesh and CRP have been conducted in Western Countries, where red meat is thought to drive this association with inflammation. However, it is important to note some distinct characteristics of the dietary profile of our study population. Unlike studies from Western countries, mutton is the primary source of red meat in our study population. Furthermore, the animal flesh intake profile is distinct in our study population, where overall animal flesh intake (74.4 g/day), and especially red meat intake (37.7 g/day), is lower compared to other general populations in Western countries. For example, daily meat intake among the US population is 127.9 g/day [33] and that among the Swedish population is 145g/day [34].

Our results also indicate that higher intakes of animal flesh, particularly poultry intake, were associated with lower concentrations of I-FABP. I-FABP is expressed in epithelial cells of the intestine tissue layer, and increased circulating concentrations of I-FABP indicate impaired gut barrier integrity [52]. When the intestinal integrity is compromised, bacteria translocate across the gut into circulation, ultimately leading to systemic inflammation [53]. We have previously shown that higher concentrations of plasma I-FABP are associated with preterm birth in HIV-infected pregnant women [16]. There are limited studies of poultry intake and I-FABP, even in non-pregnant populations. One study testing the impact of different types of freeze-dried animal flesh (i.e., beef, chicken and fish) on human fecal batch cultures showed that the bacteria *Bifidobacterium,* which plays an important role in gut homeostasis, grew better in a low-fat chicken-diet group [54]. This could help explain our observed associations between higher poultry intake and lower I-FABP. Taken together, our results suggest that higher intakes of poultry could be protective against gut barrier dysfunction and may reduce the risk of adverse pregnancy outcomes. One point to note is that our primary analysis with animal flesh excluded individuals with no animal flesh intake (e.g., vegetarians, and non-vegetarians who did not have any animal flesh intake). In exploratory analysis, we did not see a difference in inflammatory markers by vegetarian status, but our power was limited considering the small number of vegetarians. Given these limitations, these findings will need to be further assessed in larger studies.

Poultry and seafood intake were associated with lower concentrations of IL-13 in our pregnant population. IL-13 is a cytokine produced by Th2 cells and plays an important role in inflammation related to asthma, atopic dermatitis and atopic eczema [55,56,57]. Results from studies in young children have shown that fish consumption decreases the risk of atopic eczema [58,59] but the reasons for this are not clear. A population-based mother and child cohort study showed that higher intakes of all types of meat, including processed meat but not fish and seafood, during pregnancy were associated with increased risk of wheeze and eczema in the first year of life in infants [14], indicating that this association between meat and processed meat intake and risk of eczema was driven more by red meat intake rather than seafood or poultry intake. Our findings suggest that larger studies should specifically address the relationship of seafood, red meat and poultry intake with IL-13 and Th2 cytokines in pregnant populations.

The strengths of our study include the assessment of relationships of vegetable and animal flesh intake, along with the sub-categories of types of vegetables or animal flesh, with markers of inflammation informing on different aspects of immunity. Many of these observed associations with specific markers are novel, especially in a non-Western pregnant population. In addition, our results were robust to adjustments for important covariates such as HIV, LTBI and gestational diabetes. Our study has limitations: Although we were able to detect multiple associations that were significantly different, we had a relatively small sample size (n = 186) which could have limited our ability to detect other potential associations. Due to the limited sample size and because we had a specific hypothesis concerning higher vegetable and lower animal flesh intake being associated with lower concentrations of specific markers (e.g., sCD163, CRP, I-FABP), we did not adjust for multiple comparisons. We should note that some of the other associations were more exploratory (e.g., IL-17, IL-13; sub-groups of dietary factors) and hypothesis-generating. Future larger studies that either specifically focus on some of these markers or are powered to account for multiple comparisons will need to confirm our findings. Another limitation of our study is that our results presented with regression coefficient units are not easily interpretable in terms of dietary intake; however, these findings on vegetable and animal flesh intake primarily serve to better understand the relationships with specific markers and will need to be followed up in future studies to understand the impact on clinical outcomes. Lastly, relationships of diet and inflammation were only evaluated during the third trimester.

## 5. Conclusions

In conclusion, our study fills gaps in the literature regarding vegetable and animal flesh intake and their association with various aspects of systemic immunity. Our data suggests that a diet with higher intakes of vegetables, and higher intakes of poultry and seafood in a low animal flesh intake population, is associated with a reduction of monocyte/macrophage activation, improved intestinal integrity and lower levels of Th2 and Th17 cytokines. Further studies are needed to confirm these findings, expand on potential mechanisms and assess causality by testing whether modulating vegetable and animal flesh intake can impact perinatal health outcomes.

## Figures and Tables

**Table 1:** Characteristics of the study population (*N* = 186).

	Overall
	*N* (%)
Age, median (range)	24 (18–40)
Income	
≤Rs. 10,255	64 (34.9)
>Rs. 10,255	120 (65.1)
Education	
None to primary	43 (23.1)
Middle school to high school	119 (63.9)
Post high school	24 (12.9)
Mid-upper arm circumference	
<23 cm	54 (29.0)
23–30.5 cm	117 (62.9)
>30.5 cm	15 (8.1)
Current Smoker	
Yes	21 (11.3)
No	165 (88.7)
Gestational age at sampling, median (IQR)	29.3 (28.5–30.6)
Preeclampsia	
Yes	21 (11.3)
No	165 (88.7)
Gestational Diabetes status	
Gestational diabetes	18 (10.1)
No gestational diabetes	165 (89.9)
LTBI	
Yes	131 (70.4)
No	55 (29.6)
HIV	
Yes	58 (31. 2)
No	128 (68.8)
Vegetarian	
Yes	23 (12.4)
No	163 (87.6)

Legend: Data are presented as number (%) of subjects unless otherwise stated. Abbreviation: IQR, interquartile range; HIV, human immunodeficiency virus; LTBI, latent tuberculosis infection; Rs. Rupees

**Table 2:** Daily food groups intake of the study population.

(grams/day)	*N*	Median	IQR
Total vegetables and fruits	186	432.0	(322.5, 602.5)
Total vegetables	186	324.0	(244.4, 450.5)
Dry vegetables	186	185.7	(135.0, 260.3)
Green vegetables	186	76.7	(50.0, 105.5)
Raw vegetables/Salad	186	57.6	(36.0, 85.7)
Fruits	186	79.8	(27.4, 180.4)
Animal flesh (red meat + poultry + seafood) ^1^	157	74.4	(45.4, 135.8)
Red Meat	99	37.7	(17.6, 50.3)
Poultry	150	47.0	(23.5, 50.4)
Seafood	113	31.5	(13.7, 54.2)

Abbreviation: IQR, interquartile range. ^1^ Intakes are limited to those who reported any consumption during the reference period.

## Supplementary Materials

The following are available online at https://www.mdpi.com/2072-6643/12/12/3767/s1, Table S1: Different types of food items within each food group; Table S2: Association of total vegetable and fruit intake during pregnancy with inflammation in the third trimester; Table S3: Association of total vegetable intake during pregnancy with inflammation in the third trimester; Table S4: Association of fruit intake during pregnancy with inflammation in the third trimester; Table S5: Association of animal flesh (red meat + poultry + seafood) intake during pregnancy with inflammation in the third trimester; Table S6: Association of dry vegetable intake during pregnancy with inflammation in the third trimester; Table S7: Association of green vegetable intake during pregnancy with inflammation in the third trimester; Table S8: Association of salad intake during pregnancy with inflammation in the third trimester; Table S9: Association of red meat intake during pregnancy with inflammation in the third trimester; Table S10: Association of poultry intake during pregnancy with inflammation in the third trimester; Table S11: Association of seafood intake during pregnancy with inflammation in the third trimester.
