# Supplementary material for: Association of Vegetable and Animal Flesh Intake with Inflammation in Pregnant Women from India
Source: Nutrients. 2020 Dec 8;12(12):3767. doi: 10.3390/nu12123767 (PMC7762525; doi:10.3390/nu12123767)
Supplement: Supplementary file 1 [file nutrients-12-03767-s001.pdf]

Supplementary Materials: The following are available online at www.mdpi.com/xxx/s1, Table S1-S9.

Supplementary Table 1: Different types of food items within each food group

Food group	Food Names
**Dry vegetables**	Eggplant, cauliflower, sheng drumstick, peas/vatana, potato, gavar, okra, bottle gourd, flat beans, French beans, yam, tondali bitter gourd, tinda, snake gourd, pumpkin, ridge gourd, capsicum, paneer
**Green vegetables**	cabbage, methi, spinach, shepu, radish leaves, lal mat, chawli, chuka, spring onion
**Raw vegetables/Salad**	koshimbir, raw beet root, raw carrot, onion, raw radish(white), raw cucumber, raw tomato
**Fruits**	Sapota/Chikku, musk melon/kharbooj, watermelon, guava, grapes, apple, orange, sweet lime, papaya, mango, banana, figs (anjir), pineapple, pomegranate, custard apple
**Red meat**	Mutton (shammi) kebab, mutton curry, fried mutton, liver (Kaleji), mutton paya, mutton masala, button kheema ball curry
**Poultry**	Chicken curry, tandoori chicken chicken lollypop, chilli chicken, chicken masala, chicken fry, chicken tikka, chicken kheema ball curry, pork curry
**Seafood**	Prawn curry, crab curry, dry fish curry, fresh fish fry, fresh fish curry

Legend: Individual food items under each food group

Supplementary Table 2: Association of total vegetable and fruit intake during pregnancy with inflammation in the third trimester

	Univariable model *N = 186*		Multivariable model *N = 186*	
**Third trimester**	**Coefficient (95% CI)**	**P value**	**Coefficient (95% CI)**	**P value**
**Log_2 IFNB**	-0.54 (-1.28-0.20)	0.15	-0.65 (-1.44-0.13)	0.10
**Log_2 CRP**	0.16 (-0.39 – 0.71)	0.56	0.26 (-0.32-0.84)	0.38
**Log_2 AGP**	-0.022 (-0.28-0.24)	0.86	-0.042 (-0.32-0.23)	0.76
**Log_2 I-FAPB**	-0.077 (-0.55-0.39)	0.75	-0.075(-0.57-0.42)	0.77
**Log_2 IFN-g**	-0.27 (-0.85-0.31)	0.37	-0.22(-0.83-0.39)	0.47
**Log_2 IL-1b**	0.19 (-0.39-0.78)	0.52	0.32(-0.31-0.94)	0.32
**Log_2 sCD14**	-0.12 (-0.41-0.16)	0.39	-0.12(-0.41-0.17)	0.41
**Log_2 CD163**	-0.13 (-0.36-0.11)	0.29	-0.14 (-0.39-0.11)	0.27
**Log_2 TNF-alpha**	0.19 (-0.15-0.53)	0.27	0.23 (-0.14-0.59)	0.22
**Log_2 IL-6**	0.096(-0.62-0.81)	0.79	0.026 (-0.72-0.77)	0.95
**Log_2 IL-17a**	-0.22 (-0.42-(-0.017))	0.03	-0.21 (-0.43-(-0.0001))	0.05
**Log_2 IL-13**	0.012(-0.43-0.46)	0.96	0.04(-0.43-0.51)	0.87

Legend: Using linear regression, the mean difference in log_2 concentration of each inflammation markers and 95% confidence intervals (95%CI) per unit (log_e grams/day) change in total vegetables and fruit intake in individuals is calculated. Inflammation markers were measured in samples collected at the third trimester of pregnancy. Total vegetables and fruit intake and daily energy intake are log e transformed. Univariable models are adjusted for daily energy intake. Multivariable model are adjusted for daily energy intake, HIV status, age, education, gestational age at sampling and smoking status.

Supplementary Table 3: Association of total vegetable intake during pregnancy with inflammation in the third trimester

	**Univariable model** *N = 186*		**Multivariable model** *N = 186*	
**Third trimester**	**Coefficient (95% CI)**	**P value**	**Coefficient (95% CI)**	**P value**
**Log$_2$ IFNB**	-0.34 (-1.13-0.45)	0.39	-0.46 (-1.31-0.39)	0.28
**Log$_2$ CRP**	0.26 (-0.32-0.84)	0.38	0.38(-0.25-1.00)	0.24
**Log$_2$ AGP**	-0.12 (-0.39-0.15)	0.38	-0.15(-0.45-0.14)	0.32
**Log$_2$ I-FAPB**	-0.045 (-0.55-0.46)	0.86	-0.044(-0.58-0.49)	0.87
**Log$_2$ IFN-g**	-0.48 (-1.09-0.14)	0.13	-0.46(-1.11-0.19)	0.17
**Log$_2$ IL-1b**	0.036 (-0.59-0.66)	0.91	0.14 (-0.53-0.82)	0.68
**Log$_2$ sCD14**	-0.051(-0.35-0.25)	0.74	-0.049(-0.36-0.26)	0.76
**Log$_2$ CD163**	-0.27(-0.52-(-0.031))	0.03	-0.31(-0.57-(-0.046))	0.02
**Log$_2$ TNF-alpha**	0.12 (-0.25-0.48)	0.53	0.14(-0.25-0.54)	0.47
**Log$_2$ IL-6**	0.014 (-0.75-0.77)	0.97	-0.11(-0.91-0.69)	0.79
**Log$_2$ IL-17a**	-0.25 (-0.46-(-0.033))	0.02	-0.26(-0.49-(-0.025))	0.03
**Log$_2$ IL-13**	-0.15 (-0.62-0.33)	0.54	-0.13 (-0.64-0.38)	0.62

Legend: Using linear regression, the mean difference in log$_2$ concentration of each inflammation markers and 95% confidence intervals (95%CI) per unit (log$_e$ grams/day) change in total vegetables intake in individuals is calculated. Inflammation markers were measured in samples collected at the third trimester of pregnancy. Total vegetables intake and daily energy intake are log e transformed. Univariable models are adjusted for daily energy intake. Multivariable models are adjusted for daily energy intake, HIV status, age, education, gestational age at sampling and smoking status.

Supplementary Table 4: Association of fruit intake during pregnancy with inflammation in the third trimester

	Univariable model *N = 186*		Multivariable model *N = 186*	
**Third trimester**	**Coefficient (95% CI)**	**P value**	**Coefficient (95% CI)**	**P value**
**Log_2 IFNB**	-0.14(-0.32-0.039)	0.13	-0.13(-0.31-0.047)	0.15
**Log_2 CRP**	-0.033(-0.16-0.097)	0.62	-0.023(-0.16-0.11)	0.73
**Log_2 AGP**	0.0096 (-0.052-0.071)	0.76	0.0029 (-0.06-0.065)	0.93
**Log_2 I-FAPB**	-0.006 (-0.12-0.10)	0.92	0.0023 (-0.11-0.12)	0.97
**Log_2 IFN-g**	0.0044(-0.13-0.14)	0.95	0.028(-0.11-0.17)	0.69
**Log_2 IL-1b**	-0.057(-0.20-0.082)	0.42	-0.043(-0.19-0.10)	0.55
**Log_2 sCD14**	-0.032 (-0.099-0.036)	0.35	-0.015 (-0.081-0.051)	0.66
**Log_2 CD163**	0.0046(-0.052-0.061)	0.87	0.004 (-0.054-0.062)	0.90
**Log_2 TNF-alpha**	0.032 (-0.05-0.11)	0.44	0.038 (-0.045-0.12)	0.36
**Log_2 IL-6**	-0.073(-0.24-0.096)	0.40	-0.065(-0.24-0.10)	0.45
**Log_2 IL-17a**	-0.033 (-0.08-0.015)	0.18	-0.031(-0.08-0.019)	0.22
**Log_2 IL-13**	0.014 (-0.091-0.12)	0.79	0.012 (-0.097-0.12)	0.83

Legend: Using linear regression, the mean difference in log_2 concentration of each inflammation markers and 95% confidence intervals (95%CI) per unit (log_e grams/day) change in total fruit intake in individuals is calculated. Inflammation markers were measured in samples collected at the third trimester of pregnancy. Total fruit intake and daily energy intake are log e transformed. Univariable models are adjusted for daily energy intake. Multivariable models are adjusted for daily energy intake, HIV status, age, education, gestational age at sampling and smoking status.

Supplementary Table 5: Association of animal flesh (red meat + poultry + seafood) intake during pregnancy with inflammation in the third trimester

	Univariable model *N = 157*		Multivariable model *N = 157*	
**Third trimester**	**Coefficient (95% CI)**	**P value**	**Coefficient (95% CI)**	**P value**
**Log_2 IFNB**	-0.16(-0.59-0.27)	0.47	-0.13 (-0.57-0.31)	0.57
**Log_2 CRP**	0.34(0.035-0.64)	0.03	0.33 (0.025-0.64)	0.03
**Log_2 AGP**	0.026(-0.12-0.17)	0.72	0.019 (-0.13-0.16)	0.80
**Log_2 I-FAPB**	-0.28(-0.55-(-0.005))	0.046	-0.29 (-0.57-(-0.023))	0.03
**Log_2 IFN-g**	-0.079(-0.43-0.27)	0.65	-0.024 (-0.37-0.32)	0.89
**Log_2 IL-1b**	-0.32(-0.65-0.007)	0.055	-0.33 (-0.66-0.004)	0.05
**Log_2 sCD14**	0.016(-0.14-0.17)	0.84	0.019 (-0.13-0.17)	0.80
**Log_2 CD163**	0.055(-0.08-0.19)	0.42	0.053 (-0.086-0.19)	0.45
**Log_2 TNF-alpha**	-0.075(-0.28-0.13)	0.47	-0.059 (-0.27-0.15)	0.58
**Log_2 IL-6**	-0.34(-0.73-0.05)	0.09	-0.37 (0.75-0.016)	0.06
**Log_2 IL-17a**	-0.02(-0.14-0.10)	0.73	-0.03 (-0.15-0.089)	0.62
**Log_2 IL-13**	-0.23(-0.48- 0.03)	0.08	-0.21 (-0.48-0.046)	0.11

Legend: Using linear regression, the mean difference in log_2 concentration of each inflammation markers and 95% confidence intervals (95%CI) per unit (log_e grams/day) change in animal flesh intake in individuals is calculated. Those who were vegetarians and those who consumed neither red meat, poultry or seafood were excluded for analysis. Inflammation markers were measured in samples collected at the third trimester of pregnancy. Animal flesh intake and daily energy intake are log e transformed. Univariable models are adjusted for daily energy intake. Multivariable models are adjusted for daily energy intake, HIV status, age, education, gestational age at sampling and smoking status.

Supplementary Table 6: Association of dry vegetable intake during pregnancy with inflammation in the third trimester

	**Univariable model** *N = 186*		**Multivariable model** *N = 186*	
**Third trimester**	**Coefficient (95% CI)**	**P value**	**Coefficient (95% CI)**	**P value**
**Log_2 IFNB**	0.006 (-0.67-0.68)	0.99	-0.066 (-0.76-0.63)	0.85
**Log_2 CRP**	0.045 (-0.45-0.54)	0.86	0.07 (-0.44-0.58)	0.79
**Log_2 AGP**	-0.13 (-0.36-0.11)	0.29	-0.13(-0.37-0.11)	0.29
**Log_2 I-FAPB**	-0.14 (-0.56 -0.29)	0.52	-0.15(-0.58-0.28)	0.50
**Log_2 IFN-g**	-0.087 (-0.62-0.44)	0.75	-0.13 (-0.67-0.41)	0.63
**Log_2 IL-1b**	-0.39 (-0.92-0.14)	0.15	-0.37 (-0.92-0.18)	0.19
**Log_2 sCD14**	0.10 (-0.15-0.36)	0.43	0.07 (-0.18-0.32)	0.58
**Log_2 CD163**	-0.28 (-0.49-(-0.076))	0.008	-0.29(-0.51-(-0.08))	0.007
**Log_2 TNF-alpha**	0.045 (-0.27-0.36)	0.78	0.031 (-0.29-0.35)	0.85
**Log_2 IL-6**	-0.53(-1.17-0.12)	0.11	-0.63 (-1.27-0.022)	0.058
**Log_2 IL-17a**	-0.33 (-0.51-(-0.15))	<0.001	-0.33 (-0.51-(-0.14))	0.001
**Log_2 IL-13**	-0.22 (-0.62-0.19)	0.29	-0.21(-0.63-0.21)	0.33

Legend: Using linear regression, the mean difference in log_2 concentration of each inflammation markers and 95% confidence intervals (95%CI) per unit (log_e grams/day) change in dry vegetables intake in individuals is calculated. Inflammation markers were measured in samples collected at the third trimester of pregnancy. Dry vegetables intake and daily energy intake are log e transformed. Univariable models are adjusted for daily energy intake. Multivariable models are adjusted for daily energy intake, HIV status, age, education, gestational age at sampling and smoking status.

Supplementary Table 7: Association of green vegetable intake during pregnancy with inflammation in the third trimester

	**Univariable model** *N = 186*		**Multivariable model** *N = 186*	
**Third trimester**	**Coefficient (95% CI)**	**P value**	**Coefficient (95% CI)**	**P value**
**Log_2 IFNB**	-0.39(-0.93-0.15)	0.16	-0.46(-1.04-0.11)	0.11
**Log_2 CRP**	0.37 (-0.03-0.76)	0.07	0.44 (0.023-0.85)	0.04
**Log_2 AGP**	-0.17 (-0.35-0.021)	0.08	-0.17(-0.37-0.021)	0.08
**Log_2 I-FAPB**	0.18(-0.16-0.52)	0.31	0.17(-0.18-0.53)	0.33
**Log_2 IFN-g**	-0.10(-0.53-0.32)	0.63	-0.074(-0.51-0.37)	0.74
**Log_2 IL-1b**	0.16(-0.27-0.59)	0.47	0.20(-0.25-0.65)	0.37
**Log_2 sCD14**	0.047(-0.16-0.25)	0.66	0.033(-0.17-0.24)	0.75
**Log_2 CD163**	-0.18(-0.35-(-0.010))	0.04	-0.19(-0.37-(-0.016))	0.03
**Log_2 TNF-alpha**	0.20(-0.048-0.45)	0.11	0.23(-0.034-0.49)	0.09
**Log_2 IL-6**	0.41 (-0.11-0.92)	0.12	0.35(-0.18-0.88)	0.19
**Log_2 IL-17a**	-0.049(-0.20-0.10)	0.52	-0.042(-0.20-0.11)	0.60
**Log_2 IL-13**	-0.10 (-0.43-0.22)	0.52	-0.083 (-0.42-0.26)	0.63

Legend: Using linear regression, the mean difference in log_2 concentration of each inflammation markers and 95% confidence intervals (95%CI) per unit (log_e grams/day) change in green vegetables intake in individuals is calculated. Inflammation markers were measured in samples collected at the third trimester of pregnancy. Green vegetables intake and daily energy are log e transformed. Univariable models are adjusted for daily energy intake. Multivariable models are adjusted for daily energy intake, HIV status, age, education, gestational age at sampling and smoking status.

Supplementary Table 8: Association of salad intake during pregnancy with inflammation in the third trimester

	**Univariable model** *N = 183*		**Multivariable model** *N = 183*	
**Third trimester**	**Coefficient (95% CI)**	**P value**	**Coefficient (95% CI)**	**P value**
**Log_2 IFNB**	-0.30(-0.68-0.084)	0.13	-0.33(-0.74-0.075)	0.11
**Log_2 CRP**	-0.0089(-0.29-0.27)	0.95	0.016(-0.29-0.32)	0.92
**Log_2 AGP**	0.045(-0.087-0.18)	0.50	0.045 (-0.098-0.19)	0.53
**Log_2 I-FAPB**	-0.03 (-0.27-0.21)	0.81	-0.0093 (-0.27-0.25)	0.95
**Log_2 IFN-g**	-0.30(-0.60-(-0.0061))	0.046	-0.24(-0.55-0.082)	0.15
**Log_2 IL-1b**	0.0027 (-0.30-0.31)	0.99	0.059(-0.27-0.39)	0.72
**Log_2 sCD14**	-0.06 (-0.20-0.084)	0.41	-0.012(-0.16-0.14)	0.87
**Log_2 CD163**	0.026 (-0.093-0.15)	0.66	0.037(-0.093-0.17)	0.58
**Log_2 TNF-alpha**	-0.046(-0.22-0.13)	0.60	-0.044(-0.23-0.15)	0.65
**Log_2 IL-6**	-0.006 (-0.37-0.36)	0.98	-0.039(-0.43-0.35)	0.84
**Log_2 IL-17a**	-0.058(-0.16-0.047)	0.28	-0.055(-0.17-0.057)	0.33
**Log_2 IL-13**	0.011 (-0.22-0.24)	0.92	0.012(-0.24-0.26)	0.92

Legend: Using linear regression, the mean difference in log_2 concentration of each inflammation markers and 95% confidence intervals (95%CI) per unit (log_e grams/day) change in salad intake in individuals is calculated. Inflammation markers were measured in samples collected at the third trimester of pregnancy. Salad intake and daily energy intake are log e transformed. Univariable models are adjusted for daily energy intake. Multivariable models are adjusted for daily energy intake, HIV status, age, education, gestational age at sampling and smoking status.

Supplementary Table 9: Association of red meat intake during pregnancy with inflammation in the third trimester

	Univariable model<br>*N = 99*		Multivariable model<br>*N = 99*	
**Third trimester**	**Coefficient (95% CI)**	**P value**	**Coefficient (95% CI)**	**P value**
**Log_2 IFNB**	-0.15 (-0.65-0.36)	0.57	-0.14 (-0.63-0.36)	0.59
**Log_2 CRP**	0.21(-0.096-0.51)	0.18	0.21 (-0.10-0.52)	0.19
**Log_2 AGP**	-0.0013(-0.17-0.17)	0.99	-0.0063 (-0.18-0.17)	0.94
**Log_2 I-FAPB**	-0.16(-0.49-0.16)	0.32	-0.12 (-0.44-0.21)	0.48
**Log_2 IFN-g**	0.10(-0.35-0.55)	0.65	0.16 (-0.28-0.60)	0.47
**Log_2 IL-1b**	-0.092(-0.45-0.27)	0.61	-0.058 (-0.43-0.31)	0.76
**Log_2 sCD14**	0.029 (-0.15-0.21)	0.75	0.07 (-0.10-0.24)	0.42
**Log_2 CD163**	0.021 (-0.14-0.18)	0.80	0.037 (-0.13-0.20)	0.66
**Log_2 TNF-alpha**	-0.019 (-0.28-0.24)	0.89	-0.003 (-0.27-0.26)	0.98
**Log_2 IL-6**	-0.17(-0.60-0.26)	0.44	-0.12 (-0.53-0.30)	0.59
**Log_2 IL-17a**	0.0085 (-0.13-0.14)	0.90	0.021 (-0.12-0.16)	0.77
**Log_2 IL-13**	-0.16 (-0.45- 0.14)	0.30	-0.15 (-0.46-0.15)	0.33

Legend: Using linear regression, the mean difference in log_2 concentration of each inflammation markers and 95% confidence intervals (95%CI) per unit (log_e grams/day) change in red meat intake in individuals is calculated. Those who were vegetarians and those who did not consume red meat were excluded for analysis. Inflammation markers were measured in samples collected at the third trimester of pregnancy. Red meat intake and daily energy intake are log e transformed. Univariable models are adjusted for daily energy intake. Multivariable models are adjusted for daily energy intake, HIV status, age, education, gestational age at sampling and smoking status.

Supplementary Table 10: Association of poultry intake during pregnancy with inflammation in the third trimester

	**Univariable model** *N = 150*		**Multivariable model** *N = 150*	
**Third trimester**	**Coefficient (95% CI)**	**P value**	**Coefficient (95% CI)**	**P value**
**Log_2 IFNB**	-0.033 (-0.51-0.44)	0.89	-0.0054 (-0.49-0.48)	0.98
**Log_2 CRP**	0.12(-0.21-0.46)	0.48	0.12 (-0.23-0.46)	0.50
**Log_2 AGP**	-0.043(-0.20-0.11)	0.59	-0.057 (-0.22-0.10)	0.48
**Log_2 I-FAPB**	-0.38(-0.67-(-0.085))	0.01	-0.39 (-0.69-(-0.097))	0.01
**Log_2 IFN-g**	-0.011 (-0.40-0.37)	0.95	0.064 (-0.32-0.45)	0.74
**Log_2 IL-1b**	-0.36(-0.72-0.001)	0.05	-0.35 (-0.72-0.018)	0.06
**Log_2 sCD14**	0.087 (-0.09-0.26)	0.34	0.10 (-0.067-0.27)	0.24
**Log_2 CD163**	-0.054 (-0.20-0.096)	0.48	-0.051 (-0.21-0.10)	0.52
**Log_2 TNF-alpha**	-0.15 (-0.37-0.077)	0.20	-0.13 (-0.36-0.10)	0.28
**Log_2 IL-6**	-0.38(-0.80-0.047)	0.08	-0.40 (-0.81-0.02)	0.06
**Log_2 IL-17a**	-0.085 (-0.21-0.041)	0.19	-0.087 (-0.22-0.04)	0.18
**Log_2 IL-13**	-0.33(-0.61- (-0.056))	0.02	-0.33 (-0.61-(-0.041))	0.03

Legend: Using linear regression, the mean difference in log_2 concentration of each inflammation markers and 95% confidence intervals (95%CI) per unit (log_e grams/day) change in poultry intake in individuals is calculated. Those who were vegetarians and those who did not consume poultry were excluded for analysis. Inflammation markers were measured in samples collected at the third trimester of pregnancy. Poultry intake and daily energy intake are log e transformed. Univariable models are adjusted for daily energy intake. Multivariable models are adjusted for daily energy intake, HIV status, age, education, gestational age at sampling and smoking status.

Supplementary Table 11: Association of seafood intake during pregnancy with inflammation in the third trimester

	Univariable model *N = 113*		Multivariable model *N = 113*	
**Third trimester**	**Coefficient (95% CI)**	**P value**	**Coefficient (95% CI)**	**P value**
**Log_2 IFNB**	0.043 (-0.40-0.49)	0.85	-0.032 (-0.50-0.44)	0.89
**Log_2 CRP**	-0.056(-0.35-0.24)	0.71	-0.11 (-0.43-0.20)	0.47
**Log_2 AGP**	-0.0019 (-0.14-0.14)	0.98	-0.01 (-0.16-0.14)	0.89
**Log_2 I-FAPB**	-0.20(-0.49-0.09)	0.18	-0.20 (-0.51-0.11)	0.20
**Log_2 IFN-g**	0.007 (-0.35-0.37)	0.97	0.018 (-0.36-0.39)	0.93
**Log_2 IL-1b**	-0.23(-0.55-0.09)	0.16	-0.22 (-0.57-0.12)	0.20
**Log_2 sCD14**	0.11 (-0.03-0.26)	0.12	0.11 (-0.032 -0.26)	0.12
**Log_2 CD163**	-0.13 (-0.26- 0.004)	0.06	-0.13 (-0.27-0.0089)	0.07
**Log_2 TNF-alpha**	-0.10 (-0.32-0.12)	0.37	-0.097 (-0.33-0.14)	0.42
**Log_2 IL-6**	-0.25(-0.61-0.12)	0.18	-0.29(-0.67-0.087)	0.13
**Log_2 IL-17a**	-0.072 (-0.18-0.04)	0.21	-0.097 (-0.21-0.021)	0.11
**Log_2 IL-13**	-0.37(-0.62- (-0.12))	0.004	-0.39(-0.66-(-0.12))	0.005

Legend: Using linear regression, the mean difference in log_2 concentration of each inflammation markers and 95% confidence intervals (95%CI) per unit (log_e grams/day) change in seafood intake in individuals is shown. Those who were vegetarians and those who did not consume seafood were excluded for analysis. Inflammation markers were measured in samples collected at the third trimester of pregnancy. Seafood intake and daily energy intake are log e transformed. Univariable models are adjusted for daily energy intake. Multivariable models are adjusted for daily energy intake, HIV status, age, education, gestational age at sampling and smoking status.
